# High-throughput sequencing reveals the core gut microbiota of the mud crab (*Scylla paramamosain*) in different coastal regions of southern China

**DOI:** 10.1186/s12864-019-6219-7

**Published:** 2019-11-08

**Authors:** Hongling Wei, Huan Wang, Lei Tang, Changkao Mu, Chunyu Ye, Lizhi Chen, Chunlin Wang

**Affiliations:** 1 School of Marine Science, Ningbo University, Ningbo, 315211 Zhejiang China; 2 Key Laboratory of Applied Marine Biotechnology, Ministry of Education, Ningbo University, Ningbo, 315211 Zhejiang China; 3 Agricultural and Rural Bureau, Sanmen County, Zhejiang Province China; 4 Fishery Technology Station, Sanmen County, Zhejiang Province China

**Keywords:** *Scylla paramamosain*, Core gut microbiota, Illumina MiSeq sequencing, 16S rRNA

## Abstract

**Background:**

*Scylla paramamosain* is a commercially important mud crab. The microbiota is a community that inhabits the crab intestine, and is important for physiological functional and host health.

**Results:**

*Proteobacteria*, *Firmicutes*, *Bacteroidetes*, *Tenericutes*, *Spirochaetae* and *Fusobacteria* were the dominant phyla of the 36 representative phyla. Eleven genera of the 820 representative genera were considered as core gut microbiota and were distributed in the five dominant phyla. The core genus of the *Proteobacteria* included *Arcobacter, Photobacterium, Vibrio, Shewanella* and *Desulfovibrio*. The other four phyla contained one or two genera. Male and female crab samples had two different core genera, (male samples: *Psychrilyobacter* & *Lactococcus*; female samples: *Clostridium_sensu_stricto_11* and *Candidatus_Bacilloplasma*).

**Conclusions:**

This is the first time core intestinal microbiota have been identified in crab from nine coastal regions of southern China. This study provides sequencing data related to the gut microbiota of *S. paramamosain,* and may contribute to probiotic development for *S. paramamosain* aquaculture industries.

## Background

*Scylla paramamosain* is a commercially important mud crab distributed along the coasts of southern China and other Indo-Pacific countries [1–4]. Mud crab production reached 231,467 tons in 2017 in China [5]. Currently, thanks to its richness, rapid growth, and high market value, the species is important in both fisheries and aquaculture in southern China [6–8].

The microbiota inhabits the intestine which is an important physiological functional organ in *S. paramamosain,* and is closely related to host health [9, 10]. Much research in humans has shown that the gut microbiota plays basic roles in nutrient absorption and immune function, which is beneficial to host health [11, 12]. Some pathological conditions, such as, inflammatory bowel disease [13], liver cirrhosis [14], cancer [15], obesity [16], and Type 1 Diabetes [17] appear to be caused by disruption to its normal balance. Research has shown that the gut microbiota are widely involved in organ development, nutrition, immunity and crustacean diseases [18–21]. Other gut microbiome research has shown that the health, eating habits and crustacean habitats are key to the formation of a symbiotic gut bacteria model [22, 23]. Although a close relationship between the crab and its gut microbiota is increasingly accepted, limited data are available on the gut microbiota of *S. paramamosain* from Southern Chinese coasts. As part of aquaculture development, it is crucial to develop better probiotics to facilitate *S. paramamosain* industries, by unraveling gut microbial composition.

In this study, Illumina MiSeq sequencing of 16S rRNA was used to identify gut microbial composition in *S. paramamosain*. Samples from southern Chinese coasts were compared to characterize core gut microbiota.

## Results

### Data summary

After filtering low-quality reads, trimming the longer homopolymer runs, adapters, barcodes and primers, and rarefying datasets, 21,993 (sample YJ-M) to 28,377 (sample XP-F) valid contigs were collected from each region, resulting in a total of 472,782 valid contigs from the nine regions. All valid contigs were delineated into OTUs using 97% sequence similarity thresholds, consistent with other studies performing deep sequencing methods [24]. A total of 2552 OTUs were obtained. Each region sample contained 87 (sample XP-F) to 755 (sample HL-M) OTUs, which differed no significantly in most of the male crabs samples than the female crabs samples (Table 1). Nevertheless, HL and ST crabs generated significant differences between female and male samples (HL: *P* = 0.0041, ST: *P* = 0.0037).

**Table 1:** Overview of sequencing data and alpha-diversity of samples from the nine coastal regions of southern China

Group	Valid contigs	OTU	Shannon	Simpson	Chao 1	Good’s coverage
HL-F	26,157 ± 1969	215 ± 57	3.48 ± 0.216	0.84 ± 0.030	338 ± 125.87	0.995 ± 0.0016
HL-M	26,168 ± 2337	755 ± 148	5.44 ± 0.993	0.91 ± 0.021	858 ± 109.83	0.988 ± 0.0008
HP-F	27,904 ± 860	154 ± 53	3.42 ± 0.495	0.80 ± 0.040	189 ± 52.33	0.997 ± 0.0008
HP-M	26,118 ± 2418	453 ± 280	4.29 ± 1.123	0.88 ± 0.070	527 ± 317.45	0.992 ± 0.0047
SM-F	28,219 ± 821	286 ± 176	4.57 ± 2.047	0.89 ± 0.080	318 ± 150.82	0.997 ± 0.0014
SM-M	27,103 ± 1061	270 ± 113	3.41 ± 0.367	0.79 ± 0.066	353 ± 146.69	0.994 ± 0.0025
RA-F	27,495 ± 1216	318 ± 193	4.79 ± 1.872	0.90 ± 0.079	385 ± 133.53	0.996 ± 0.0015
RA-M	25,757 ± 2074	179 ± 43	3.85 ± 0.594	0.86 ± 0.075	213 ± 65.40	0.997 ± 0.0011
ST-F	26,952 ± 1164	174 ± 66	3.09 ± 1.118	0.75 ± 0.143	248 ± 107.24	0.996 ± 0.0015
ST-M	27,729 ± 2215	429 ± 30	4.84 ± 1.043	0.92 ± 0.045	596 ± 91.95	0.991 ± 0.0022
TS-F	22,555 ± 3723	530 ± 242	3.55 ± 0.896	0.76 ± 0.075	617 ± 269.36	0.990 ± 0.0044
TS-M	26,197 ± 1560	186 ± 97	3.55 ± 0.579	0.84 ± 0.071	266 ± 170.60	0.996 ± 0.0030
XP-F	28,377 ± 698	87 ± 14	2.47 ± 0.471	0.70 ± 0.068	129 ± 57.67	0.998 ± 0.0006
XP-M	26,811 ± 1415	92 ± 43	2.43 ± 0.984	0.66 ± 0.217	153 ± 103.13	0.998 ± 0.0015
YJ-F	26,447 ± 657	594 ± 368	5.12 ± 2.241	0.89 ± 0.086	695 ± 394.79	0.991 ± 0.0054
YJ-M	21,993 ± 5211	378 ± 80	5.52 ± 1.623	0.92 ± 0.077	394 ± 70.29	0.998 ± 0.0016
YX-F	25,662 ± 2052	219 ± 186	4.74 ± 1.923	0.91 ± 0.066	242 ± 167.04	0.998 ± 0.0004
YX-M	25,138 ± 6631	480 ± 8	6.98 ± 0.149	0.98 ± 0.003	487 ± 15.34	0.998 ± 0.0004

The characterization of bacterial community richness, diversity and sequencing depth was performed using the alpha diversity index (Table 1). The Chao1 indices, which ranged from 129 ± 57.67 to 858 ± 109.83, were used to determine bacterial community richness in *S. paramamosain*. There were no differences in Shannon and Simpson indices. The Good’s coverage estimator of the samples ranged from 0.990 to 0.998 (Table 1), indicating that sequencing depths covered all species in samples. Meanwhile, the sparse curve reaches the saturation platform (Additional file 1: Figure S1A), manifesting that the sequencing depth is large enough to obtain a stable and unbiased estimate of species richness. In addition, the specaccum accumulation curves tend to gradually, indicating that the sample size is sufficient to reflect the abundance of the community richness, the results reflect the rate of increase in new species observed as the sample size continues to increase during the overall sampling of the sample. The number of OTUs increased rapidly from 1 to 54 and began to level off at the end of our sampling, indicating that bacterial diversity was largely saturated (Additional file 1: Figure S1B).

### Composition of microbial communities in *S. paramamosain*

At the taxonomic level, six different patterns of intestinal microbial composition were distinguished. As shown (Additional file 1: Table S1), the number of taxonomic units detected in each region were present. The pattern of gut microbial composition in male samples was greater than female samples (Additional file 1: Table S1). Additional file 1: Figure S2-S4 show microbial community composition at Class, Order, and Family levels.

**Fig. 1:**
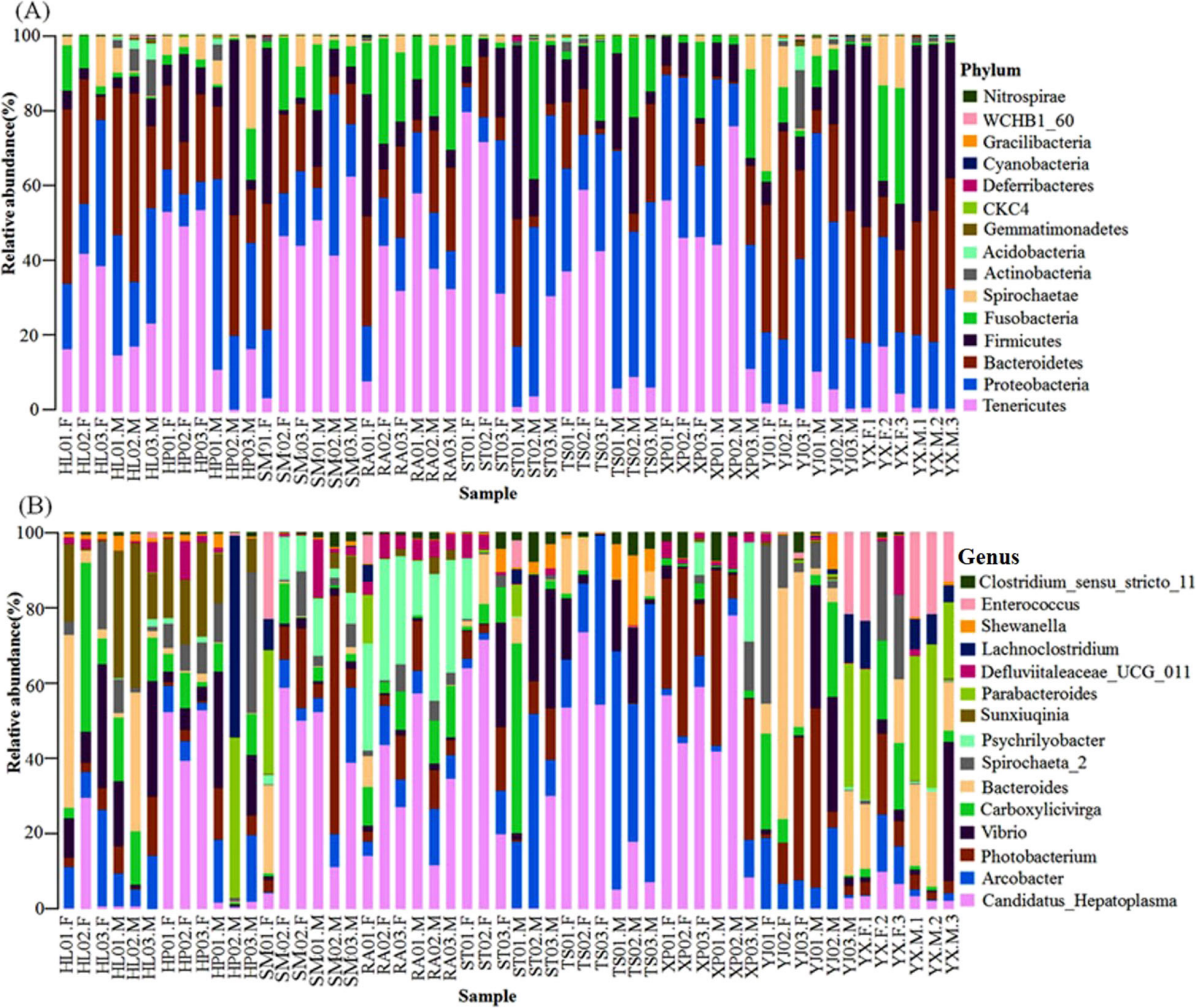
The 15 most abundant phyla and genera. **a** Bar-plots showing the abundance and distribution of the 15 most abundant phyla. **b** Bar-plots showing the abundance and distribution of the 15 most abundant genera

We found 36 different phyla (Additional file 1: Table S1) in all samples. There were differences between male and female samples (30 phyla in female samples and 35 phyla in male samples). Figure 1a and Additional file 1: Table S2 show the top 15 highly abundant phyla. *Tenericutes*, *Proteobacteria*, *Bacteroidetes*, *Firmicutes* and *Fusobacteria* were identified in all samples, another ten phyla were only detected in one or several samples, including *Spirochaetae*, *Actinobacteria*, *Acidobacteria*, *Gemmatimonadetes*, *CKC4*, *Deferribacteres*, *Cyanobacteria*, *Gracilibacteria*, *WCHB1_60* and *Nitrospirae*. *Proteobacteria* levels were significantly different between female and male samples (*P* = 0.0085). *Tenericute* levels were significantly different between female and male samples (*P* = 0.0346). No significant differences were found in the other thirteen phyla.

At the genus level, sequences from samples represented 820 genera (Additional file 1: Table S1). Genera in male samples numbered 45 more than in female samples (711 genera in female samples and 756 genera in male samples). The top 15 genera are listed in Fig. 1b and Additional file 1: Table S3; *Candidatus_Hepatoplasma*, *Arcobacter, Photobacterium*, *Vibrio*, *Carboxylicivirga*, *Bacteroides*, *Spirochaeta_2*, *Psychrilyobacter*, *Sunxiuqinia*, *Parabacteroides*, *Defluviitaleaceae_UCG_011*, *Lachnoclostridium*, *Shewanella*, *Enterococcus*, *and Clostridium_sensu_stricto_11*. These 15 genera accounted for nearly half or more of the total sequences in the samples. *Candidatus_Hepatoplasma* and *Shewanella* abundance differed significantly in female samples when compared to male samples (*P* = 0.0286, *P* = 0.0291, respectively). No significant differences were found for the other thirteen genera.

### Core gut microbiota at the genus level in *S. paramamosain*

**Fig. 2:**
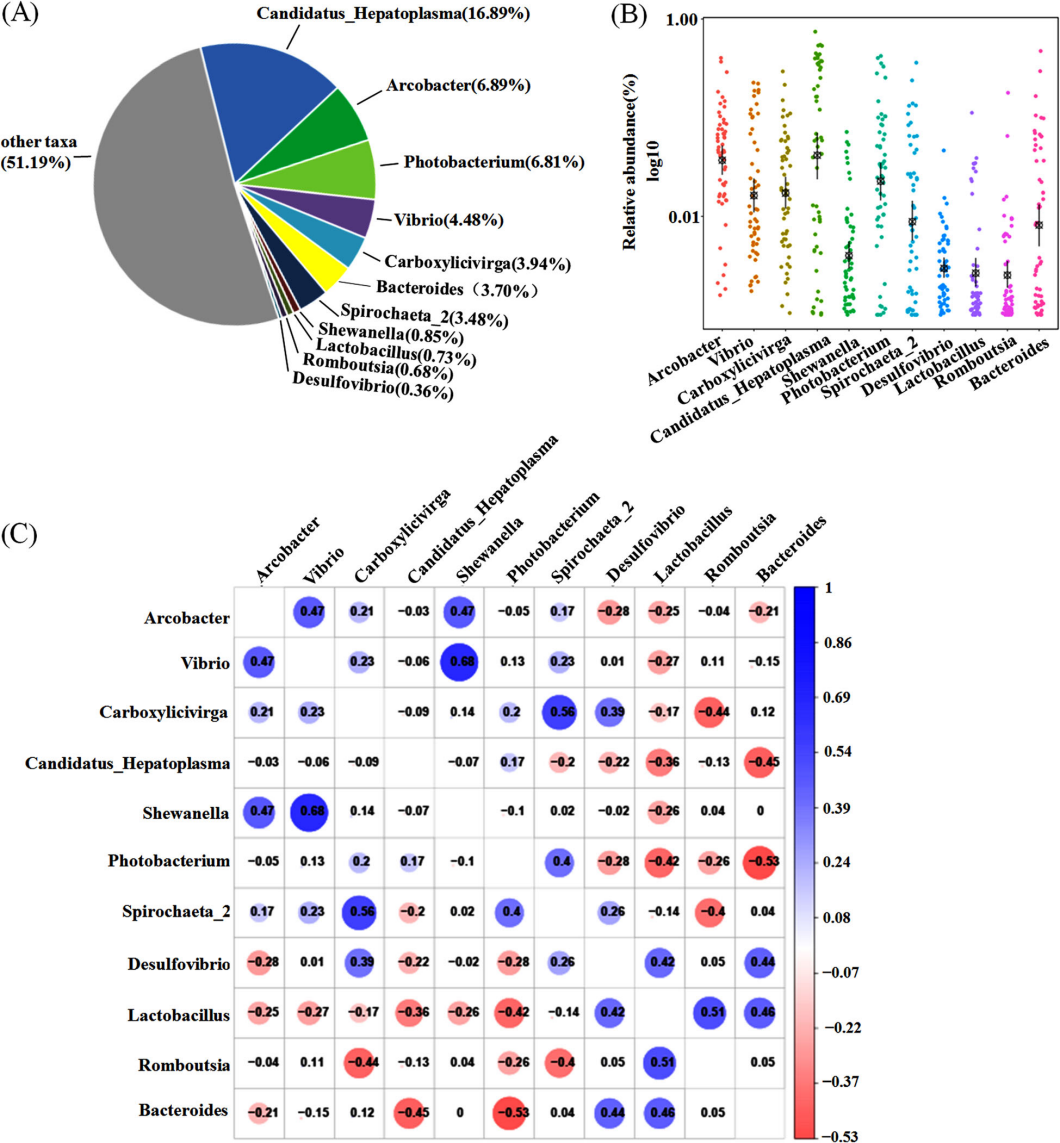
Core gut microbiota composed of 11 bacterial genera in *S. paramamosain* in nine regions samples. **a** The proportion of each genus in all sequences combined. **b** The abundance and distribution of 11 core genera. **c** Correlation matrix showing the Spearman’s rank correlations among the collective core, which ranges from − 1 to 1, corresponding to a strongly positive to a strongly negative correlation, respectively

A major goal of the study was to determine whether a common core microbiota was shared among all samples. At the genus level, we assigned 11 genera candidates (Table 2), each of these candidates exhibited a frequency of occurrence higher than 90% over all samples and were treated as core gut microbiota [25, 26]. We analyzed 11 core genera and found that these organisms constituted a phylogenetic core of the genera, accounting for 48.81% of all sequences (Fig. 2a). These 11 core genera were distributed among five phyla, and 63.6% of these genera were in the *Proteobacteria* and *Firmicutes*, with the remaining in the *Bacteroidetes*, *Tenericutes* and *Spirochaetae* genera. However, from this study, the relative abundance of these core genera varied greatly across samples (Fig. 2b and Table 2).

**Table 2:** The core genera identified in samples

Phylum	Genus	Relative abundance (%)	Range (%)
Tenericutes	Candidatus_Hepatoplasma	16.89	0.000–74.325
Proteobacteria	Arcobacter	6.89	0.058–40.186
Proteobacteria	Photobacterium	6.81	0.000–42.027
Proteobacteria	Vibrio	4.48	0.074–22.604
Bacteroidetes	Carboxylicivirga	3.94	0.004–29.438
Bacteroidetes	Bacteroides	3.70	0.000–47.259
Spirochaetae	Spirochaeta_2	3.48	0.000–35.887
Proteobacteria	Shewanella	0.85	0.000–7.066
Firmicutes	Lactobacillus	0.73	0.000–11.112
Firmicutes	Romboutsia	0.68	0.000–17.830
Proteobacteria	Desulfovibrio	0.36	0.000–4.543

We also investigated these intergeneric the co-occurrence patterns of these genera based on Spearman’s rank correlations (Fig. 2c). We observed that the genus *Candidatus_Hepatoplasma* was inversely associated with almost every other genera (Spearman’s rank correlation coefficients (ρ) ranged from − 0.45 to 0.17) and *Bacteroides* showed relatively strong negative correlations with *Photobacterium* (ρ = − 0.53). *Vibrio* was positively associated with other genera besides *Candidatus_Hepatoplasma*, *Lactobacillus* and *Bacteroides* (Spearman’s rank correlation coefficients (ρ) ranged from − 0.27 to 0.68). Other genera were positively or inversely correlated with each other to different degrees.

**Fig. 3:**
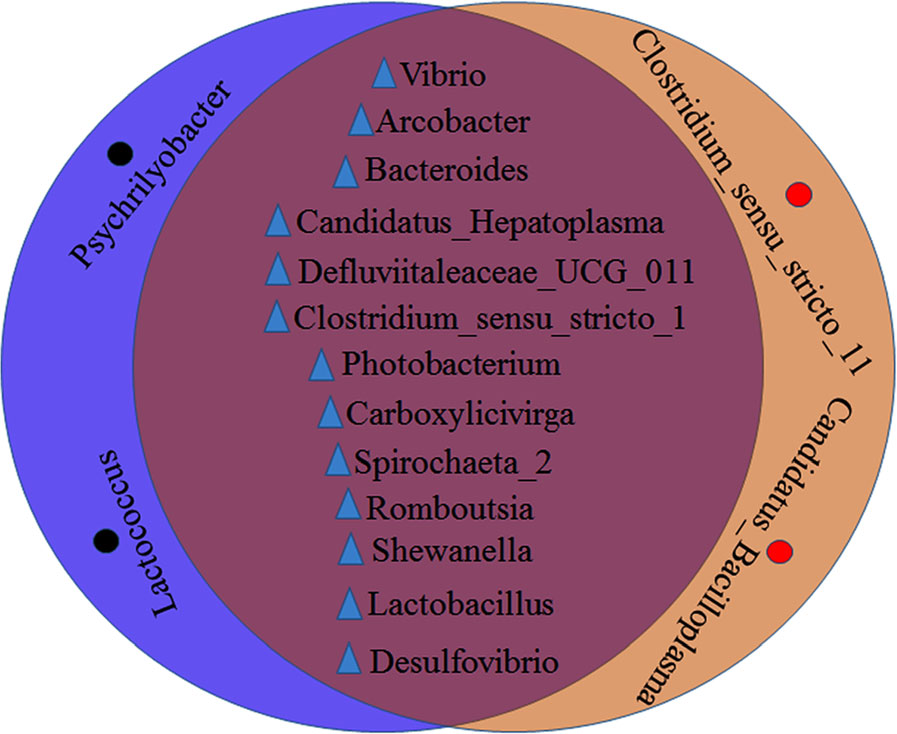
Core genera identified in male and female samples

We also found genus level differences, between male and female samples, in the core gut microbiota. Fifteen core genera were distributed between male and female samples, (Tables 3 and 4), and thirteen uniform core genera were distributed between male and female samples (Fig. 3). There were two additional core genera in female samples (*Clostridium_sensu_stricto_11* & *Candidatus_Bacilloplasma*), and two special core genera in male samples (*Psychrilyobacter* & *Lactococcus*).

**Table 3:** The core genera identified in female samples

Phylum	Genus	Relative abundance (%)	Range (%)
Tenericutes	Candidatus_Hepatoplasma	22.71	0.000–54.672
Proteobacteria	Photobacterium	6.12	0.000–40.127
Proteobacteria	Arcobacter	5.88	0.083–28.949
Bacteroidetes	Bacteroides	5.55	0.000–47.259
Bacteroidetes	Carboxylicivirga	4.78	0.206–29.438
Spirochaetae	Spirochaeta_2	4.54	0.000–35.887
Proteobacteria	Vibrio	3.05	0.074–18.715
Firmicutes	Defluviitaleaceae_UCG_011	1.59	0.000–7.729
Firmicutes	Clostridium_sensu_stricto_1	0.70	0.000–4.331
Firmicutes	Clostridium_sensu_stricto_11	0.52	0.000–6.029
Firmicutes	Romboutsia	0.41	0.000–6.419
Proteobacteria	Shewanella	0.39	0.007–3.808
Firmicutes	Lactobacillus	0.37	0.000–3.316
Proteobacteria	Desulfovibrio	0.29	0.007–1.440
Tenericutes	Candidatus_Bacilloplasma	0.24	0.000–1.511

**Table 4:** The core genera identified in male samples

Phylum	Genus	Relative abundance (%)	Range (%)
Tenericutes	Candidatus_Hepatoplasma	11.07	0.007–74.325
Proteobacteria	Arcobacter	7.91	0.058–40.186
Proteobacteria	Photobacterium	7.51	0.000–42.027
Proteobacteria	Vibrio	5.90	0.098–22.604
Fusobacteria	Psychrilyobacter	3.19	0.000–21.912
Bacteroidetes	Carboxylicivirga	3.09	0.004–14.964
Spirochaetae	Spirochaeta_2	2.42	0.000–23.831
Bacteroidetes	Bacteroides	1.84	0.000–16.469
Firmicutes	Defluviitaleaceae_UCG_011	1.47	0.000–12.943
Proteobacteria	Shewanella	1.32	0.000–5.223
Firmicutes	Lactobacillus	1.10	0.004–11.112
Firmicutes	Romboutsia	0.94	0.000–17.830
Firmicutes	Lactococcus	0.70	0.000–3.718
Firmicutes	Clostridium_sensu_stricto_1	0.60	0.000–4.932
Proteobacteria	Desulfovibrio	0.43	0.000–4.543

### Gut microbiota relationships across *S. paramamosain,* in nine regions

**Fig. 4:**
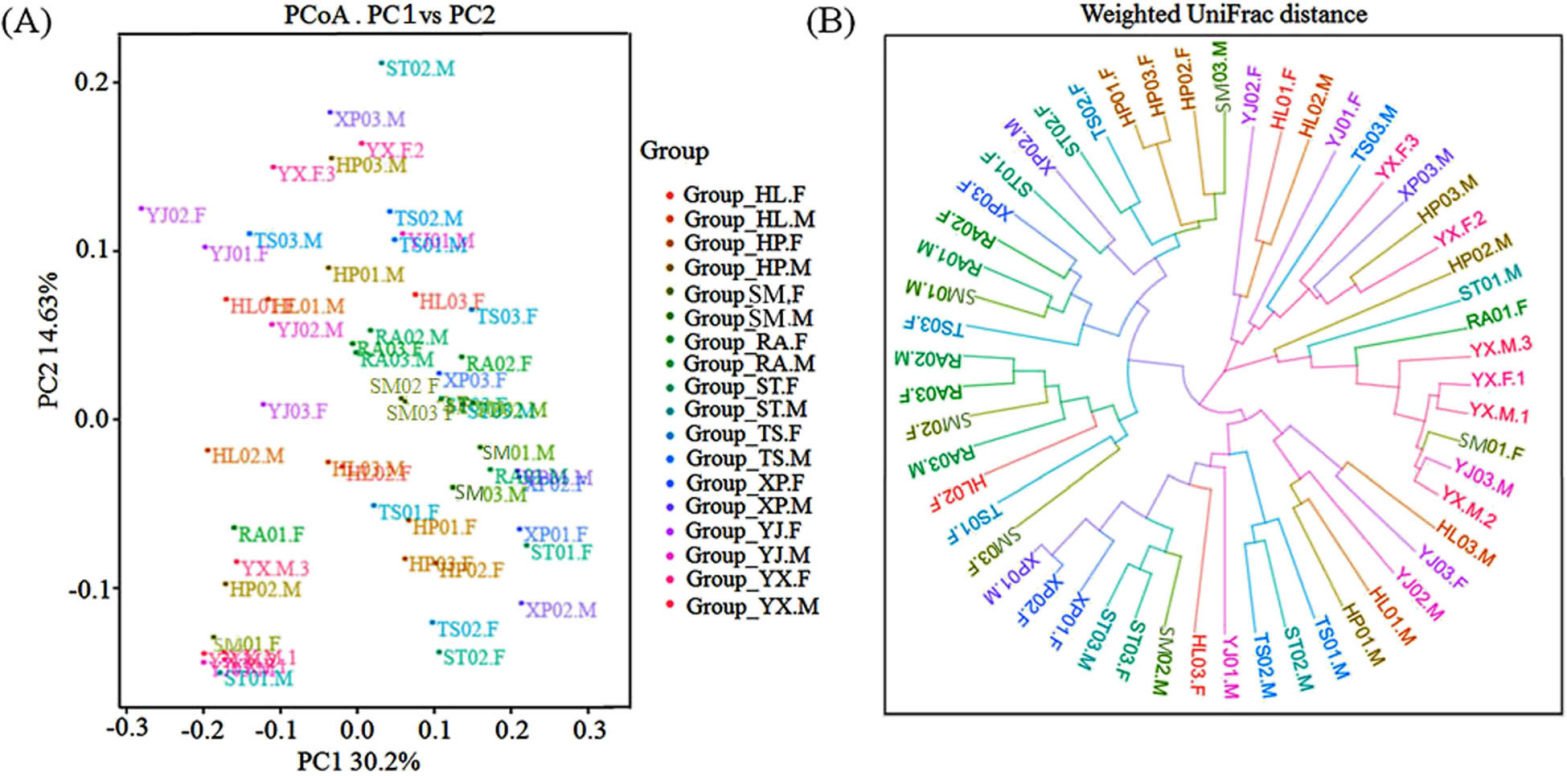
Principal coordinate analysis and circular tree plot of all samples using the weighted UniFrac distance matrices. **a** Principal coordinate analysis of the microbial communities in all samples; **b** Circular tree plot of all samples using the weighted UniFrac distance matrices

**Fig. 5:**
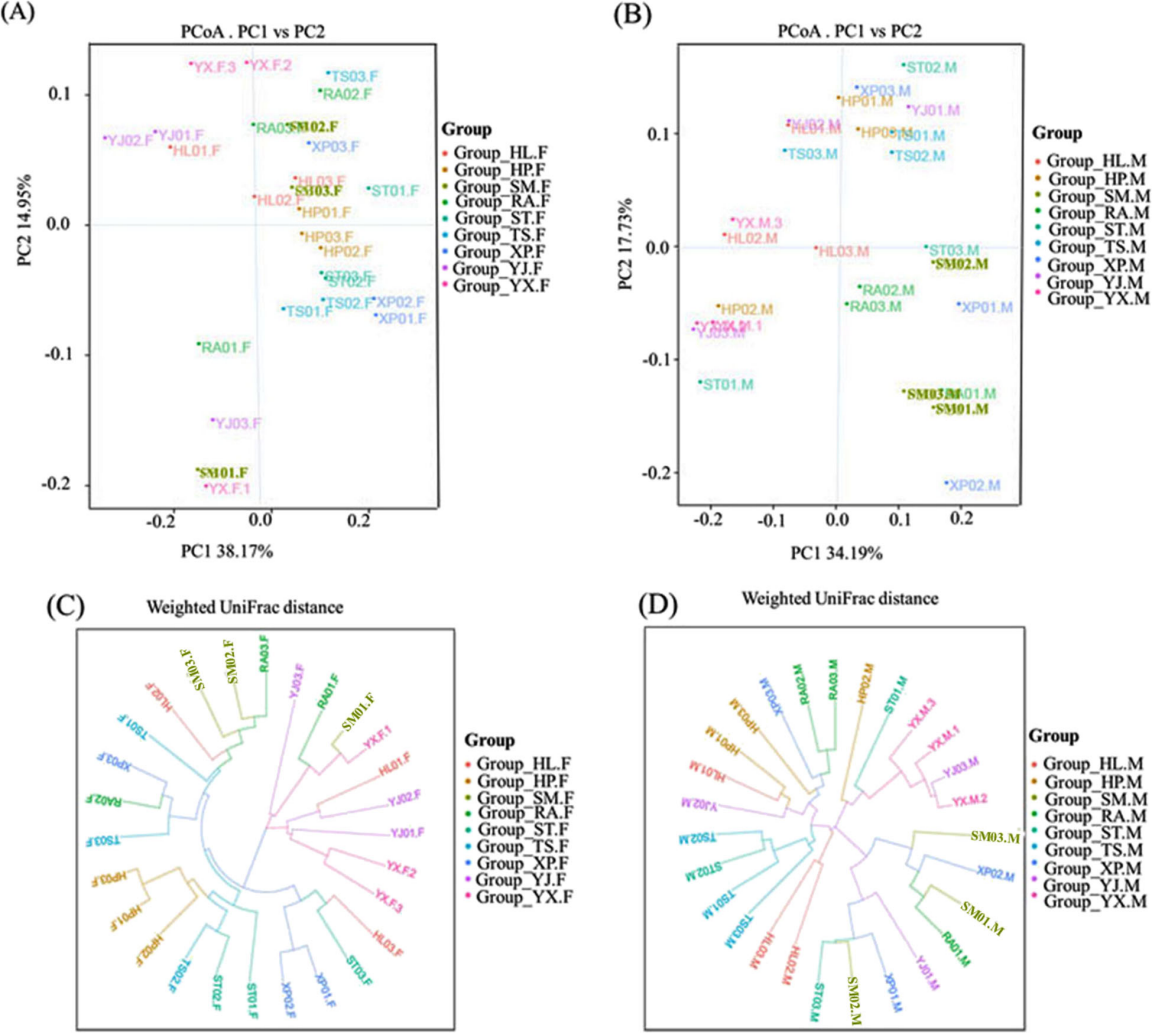
Principal coordinate analysis and circular tree plot of female or male samples using the weighted UniFrac distance matrices. **a** Principal coordinate analysis of the microbial communities in female samples; **b** Principal coordinate analysis of the microbial communities in male samples; **c** Circular tree plot of female samples using the weighted UniFrac distance matrices; **d** Circular tree plot of male samples using the weighted UniFrac distance matrices

This study investigated relationships of gut microbial communities in 54 samples using weighted UniFrac PCoA and hierarchical dendrogram analyses (Fig. 4). There were obvious separated and overlapped samples for each of the regions. Composition of the community in the nine regions, had changed. Among these, samples derived from SM, RA, XP and YX were grouped closer than the other five flocks. However, there were differences between male and female samples in community compositions. Samples HP-F, ST-F, TS-F, XP-F, YJ-F and YX-F clustered closer than samples HL-F, SM-F and RA-F. Furthermore, samples HF-M, SM-M, TS-M and YX-M were closer than samples HP-M, RA-M, ST-M, XP-M and YJ-M in term of community composition (Fig. 5a and b). UPGMA clustering analyses, based on weighted UniFrac distances, also indicated a similarly discriminative structural separation between male and female samples (Fig. 5c, d).

## Discussion

*S. paramamosain* are usually cultured in brackish, seawater ponds along the coasts of southern China and other Indo-Pacific countries. It is a commercially important mud crab distributed [3, 27]. Breeding of *S. paramamosain* mainly occurs along the coasts of southern China (Additional file 1: Figure S1), including Zhejiang, Fujian, Guangdong, Guangxi, Hainan province. Although gut microbiota regulates many aspects of digestive function, nutrition, metabolism, fat storage and gut-associated mucosal immunity [28], little is known about gut bacterial community structures in *S. paramamosain*. Hence, this study sought to examine gut microbial diversity and core gut microbiota of *S. paramamosain* from nine coastal regions in southern China. To the best of our knowledge, this study is the first to characterize core gut microbiota from *S. paramamosain* from southern Chinese coasts using state of the art, Illumina MiSeq sequencing methodologies.

Analysis of gut microbiota composition demonstrated that the dominant bacteria of the fifty-four samples belonged to six phyla, *Proteobacteria*, *Firmicutes*, *Bacteroidetes*, *Tenericutes*, *Spirochaetae* and *Fusobacteria*, and the first four phyla were also found in the *Eriocheir sinensis* gastrointestinal tract [29]. These results were consistent with a previous study on gut bacterial assemblages of *Eriocheir sinensis* from Lake Tai (286 km from Lake Gucheng in China) [30]. These dominant genera may play major roles in gut function or adapt to the environment by the digestive tract.

The 11 core genera constituted a phylogenetic core of the genus, accounting for 48.81% of total sequences. Among them, *Tenericutes* from the genus *Candidatus_Hepatoplasma*, accounted for the greatest average relative abundance. Previous research had discovered that isopods with intestinal tract based *Candidatus_Hepatoplasma*, had higher survival rates when food was deficient [31]. However, this has not yet been reported in *S. paramamosain*. In China, artificially cultured crabs are located in ponds, with little phytoplankton or zooplankton. Similarly, breeding densities are higher. In addition, farmers feed crabs at fixed times, therefore, *S. paramamosain* may be in hungry environments for prolonged periods. So, we speculated that the reason the mud crab could be able to adapt to thehunger environment is the regulation of Candidatus_Hepatoplasma. However, this conjecture must be corroborated by further research.

The core genera; *Arcobacter*, *Photobacterium*, *Vibrio*, *Shewanella* and *Desulfovibrio* belong to *Proteobacteria*. The genus *Arcobacter* is common in many marine invertebrates, such as crabs [32], mussels [33], abalones [34], and oysters [35]. The genus *Photobacterium*, which is one of the nine genera in the family *Vibrionaceae* (order “*Vibrionales*”, class *Gammaproteobacteria*), is the largest genera after *Vibrio* [36, 37]. Some of its species exhibit bioluminescence and pathogenesis mechanisms [38], with one study reporting that *Photobacterium* is a potential freshwater fish pathogen [39]. Worldwide, *Vibrio* is widely distributed in aquatic environments. However, many *Vibrio* members are considered primary pathogens in causing disease and death in aquaculture animals [40], and they seriously jeopardize the development of aquaculture. Many studies have shown that *Vibrio* provides a benefit to the host, for example, Asfie et al.*,* [41] isolated multiple strains of Protease-producing bacteria from the gingiva intestinal tract, and showed that some proteases secreted by *Vibrio* are beneficial to gums, growth and development. Similarly, Hamid et al.*,* [42] observed that *Vibrio* secreted amylases, proteases, lecithinases and chitinases to help digest important nutrients such as fat, proteins and carbohydrates in the host body. Further research also showed that *Vibrio* was present in both healthy and diseased *S. paramamosain* [27]. We therefore speculated that *Vibrio* may digest important nutrients such as fat, protein and carbohydrates in the host body by secreting amylases and proteases to maintain normal activities in healthy crabs. Therefore, it was not surprising that *Vibrio* was found in samples in this study. Our study has also illustrated the diversity of *Vibrio* and its beneficial role as a dominant bacteria in the intestine. The separation and identification of beneficial *Vibrio* species may promote crab aquaculture production.

*Firmicutes* are often found in the gut of marine invertebrates, such as sea squirt (*Ciona intestinalis*) [43], black tiger shrimp (*Penaeus monodon*) [44] and the Atlantic blue crab (*Callinectes sapidus*) [32]. The *Lactobacillus* genus belongs to the *Firmicutes*, which are commonly found in the gastrointestinal tracts of humans and other animals. In this study, *Lactobacillus* was also found in these nine regions, therefore, we speculated it may have potential probiotic properties in *S. paramamosain*. Studies have shown that due to its relevance in industrial applications in certain species, such as L. lactis, the central metabolic pathway of this genus has been extensively studied. These bacteria can convert large hexose sugar substrates to pyruvate via glycolysis and then to lactate [45]. *Lactococcus* is the focus of intensive research in carbohydrate catabolism, the industrial fermentation process [46] and its role in promoting health, such as the prevention and protection of diarrhea and intestinal infections, are important for a well-balanced gut microbiota [47, 48]. So, on one hand, due to it can prevent and protect diarrhea and intestinal infections, it is not surprising that it can be found in all samples from nine regions. On the other hand, it is worthy of further study to isolate and characterize the functional bacteria of this genus from intestinal samples, and it may develop probiotics for the *S. paramamosain* breeding industries. Moreover, the genus *Bacteroides* from the *Bacteroidetes* phyla has been associated with animal protein metabolism, a variety of 354 Yang et al., amino acids and saturated fats [49]. And other core genera of *Shewanella*, *Desulfovibrio*, *Romboutsia*, *Carboxylicivirga* and *Spirochaeta_2* in functional study have not yet been reported.

According to the experience of many farmers, there were differences in development processes between male and female crabs. However, there were 13 identical genera in the mid-gut population of male and female crabs (Fig. 3). This meant that gender had no significant effects on gut composition in *S. paramamosain*. This was consistent with Jin et al.*,* [50] and their study, the intestinal flora of *Eriocheir sinensis* in Yangcheng West Lake. An interesting discovery from this study was the difference between male and female samples in terms of the core gut microbiota, at the genus level. There were two different core genera in female samples (*Clostridium_sensu_stricto_11* and *Candidatus_Bacilloplasma*), and two additional core genera in male samples (*Psychrilyobacter* and *Lactococcus*). At present, the functions of these four genera have not been reported. We speculated these genera may be the reason for differences in flavors between male and female crabs. It will be interesting to determine which gut microbiota cause flavor differences. In this study, the 11 core genera were distributed in the five dominant phyla. Among these, five genera (Arcobacter, Photobacterium, Vibrio, Shewanella, Desulfovibrio) belonged to Proteobacteria, while the other four phyla contained one or two genera, such as Bacteroidetes (genus Carboxylicivirga & Bacteroides), Firmicutes (genus Lactobacillus & Romboutsia), Tenericutes (genus Candidatus_Hepatoplasma), and Spirochaetae (genus Spirochaeta_2). We speculated that these phyla and genera present in the *S. paramamosain*’ gut might have many reasons. Firstly, in terms of internal factors, in China, although the seedlings of these *S. paramamosain* have broken through the key technologies of large-scale breeding [51], the artificial breeding technology is not quite ripe yet. So mud crab seedlings are mainly derived from natural sea areas, these crabs in nine regions may be not the same source, they are the same species, *S. paramamosain,* therefore they are hereditary. We suspect that they have these common core genera that may be genetic factors.

On the other hand, environmental factors have also been reported to affect gut microbial composition [52]. Our analyses found 11 core genera in nine different regions. This observation may indicate that environmental factors do little to affect the structure of the gut microbiota of *S. paramamosain* or the lack of an effect may have been due to similar natural conditions (Additional file 1: Table S4), such as temperature, salinity, pH, dissolved oxygen (DO) and so on. It may be that these nine regions are all at suitable temperatures and salinities and they live in ponds that are separated by mud with black plastic membranes to prevent the loss of crabs.

## Conclusions

In this study, we used Illumina MiSeq sequencing to clarify the gut microbiota of composition from *S. paramamosain*. This was the first time we identified core intestinal microbiota from nine coastal regions of southern China. We analyzed 472,782 valid tags from nine regions samples, of which 2552 OTUs were identified. Our results showed that *Proteobacteria*, *Firmicutes*, *Bacteroidetes*, *Tenericutes*, *Spirochaetae* and *Fusobacteria* were the dominant phyla of the 36 representative phyla. 11 genera of the 820 representative genera were considered as core gut microbiota and were distributed in the five dominant phyla. The core genera distributed in *Proteobacteria* were the most genera, including *Arcobacter*, *Photobacterium*, *Vibrio*, *Shewanella*, *Desulfovibrio*. While other four phyla contain one or two genera. Moreover, there were differences between male and female samples in the core gut microbiota, at the genus level. There were two special genera in female samples (*Clostridium_sensu_stricto_11* and *Candidatus_Bacilloplasma*), and two special genera in male samples (*Psychrilyobacter* and *Lactococcus*). This study has generated much sequencing data related to the gut microbiota of *S. paramamosain* and may contribute to probiotic development for mud crab aquaculture industries in the future.

## Methods

### Sample collection in different areas

Mud crab breeding is mainly distributed along the coast of southern China (Additional file 1: Figure S5), including Zhejiang, Fujian, Guangdong, Guangxi, Hainan provinces. Firstly, we purchased crabs from local farmers in nine areas between May and June 2017. These crab collection areas were at Sanmen county, Taizhou city, Zhejiang province (SM, 29°06′16.81″N, 121°23′44.45E), Ruian county, Wenzhou city, Zhejiang province (RA, 27°46′42.17″N, 120°39′18.65″E), Xiapu county, Ningde city, Fujian province (XP, 26°53′6.61″N, 120°0′20.02″E), Yunxiao county, Zhangzhou city, Fujian province (YX, 23°57′29.02″N, 117°20′22.74″E), Shantou city, Guangdong province (ST, 23°21′22.19″N, 116°40′39.89″E), Yangjiang city, Guangdong province (YJ, 21°51′39.78″N, 111°58′39.04″E), Taishan county, Zhuhai city, Guangdong province (TS, 22°12′24.13″N, 113°17′50.43″E), Hepu county, Behai city, Guangxi zhuang autonomous region (HP, 21°39′48.50″N, 109°12′10.98″E), Hele county, Wanning city, Hainan province (HL, 18°54′0.52″N, 110°28′32.39″E). In these locations, we caught mud crab. Each region chose good limbs and good vitality 12 female and male crabs (body weight: 250-450 g), immediately isolated their intestinal tracts and contents and froze samples in liquid nitrogen. Samples were transported to the lab on dry ice. When extracting DNA, every three samples were mixed into one, so each region had six samples, with a total of 54. All samples were stored in sterile containers.

### DNA extraction, 16S rRNA gene amplification, and Illumina MiSeq sequencing

Genomic DNA was extracted from each sample using the DNeasy PowerLyzer PowerSoil Kit (QIAGEN, Hilden, Germany). DNA concentration and purity were assessed on 1% agarose gels. The V3-V4 region of the bacterial 16S rRNA gene was amplified using the barcode-fusion forward primer 343F (5′- TACGGRAGGCAGCAG-3′) and the reverse primer 798R (5′- AGGGTATCTAATCCT − 3′). The PCR was performed in a 30 μl volume with 15 μl of 2 × HiFi Hot Start Ready Mix, 1.0 μl of forward and reverse primers (10 μmol/L), and 50 ng of genomic DNA as template. Thermal cycling consisted of initial denaturation at 94 °C for 5 min; followed by 26 cycles of denaturation at 94 °C for 30 s, annealing at 56 °C for 30 s, elongation at 72 °C for 30 s and a final extension at 72 °C for 7 min.

The PCR products were purified using Agencourt Ampure XP beads (Beckman, CA, USA) according to the manufacturer’s instructions. 5 μl purified product were detected by 1% agarose gel electrophoresis and 1 ul purified product for concentration detection using a NanoDrop Lite spectrophotometer (Thermo Scientific). The purified DNA was then used as a template to perform a second PCR amplification using the same primer sequences and the protocol described above, however, 8 cycles were employed. After a further purification with the AMPure XP beads, the amplicon was measured concentration using NanoDrop Lite spectrophotometer. Purifed amplicons were pooled in equimolar and pairedend sequenced (2 × 300) on an Illumina MiSeq platform.

### Bioinformatic analyses

Raw sequencing data came in FASTQ format. Paired-end reads were preprocessed using Trimmomatic software [53], to detect and remove ambiguous bases (N). The software also removed low quality sequences, with average quality scores below 20, using the sliding window trimming approach. After trimming, paired-end reads were assembled using FLASH software [54]. The parameters for assembly were: 10 bp minimal overlap, 200 bp maximum overlap and a 20% maximum mismatch rate. Sequences were performed further denoising as follows: contigs with ambiguous, homologous sequences or below 200 bp were abandoned. Reads with 75% of bases above Q20 were retained. Contigs with chimeras were detected and removed. These steps were achieved using the QIIME software (version 1.8.0) [55]. Clean reads were subjected to primer sequence removal and clustering to generate operational taxonomic units (OTUs) using UPARSE software, with 97% similarity cutoff [56]. The representative read of each OTU was selected using QIIME software. All representative reads were annotated and blasted against the Silva database Version 123 (or Greengens) (16 s rDNA) using RDP classifier (confidence threshold was 70%) [57]. For alpha-diversity metrics, Observed Species, Chao1 estimator, Shannon Wiener Index, Simpson diversity index, Good’s Coverage with QIIME (Version 1.8.0) and displayed with R software (Version 2.15.3). For beta-diversity metrics, the weighted UniFrac distance matrix [58] was calculated and visualized using Principal Coordinate Analysis (PCoA) in QIIME. Biomarker discovery analysis of each taxonomic unit was performed using LeFse (version 1.0.7) [59]. All figures were generated with customized R scripts.

### Statistics

All statistical analyses were performed using SPSS (SPSS 21.0). ANOVA was also used for statistical analyses. Correlations between the core genera of gut microbiota were appraised by calculating nonparametric Spearman’s rank correlation coefficients, which were displayed in a correlation matrix. Results were considered significant at *P* < 0.05.

## Supplementary information

**Additional file 1: Table S1.** Number of bacterial taxonomic units. **Table S2.** Mean relative abundance of the 15 most abundant phyla in samples from the nine coastal regions of Southern China. **Table S3.** Mean relative abundance of the 15 most abundant genera in samples from the nine coastal regions of Southern China. **Table S4.** Salinity, temperature, pH, dissolved oxygen (DO), ammonia-nitrogen and nitrite levels of the nine regions of Southern China. **Figure S1.** Impact of sequencing depth and sampling on bacterial phylotypes. **Figure S2.** Composition of microbial communities at the Class level. **Figure S3.** Composition of microbial communities at the Order level. **Figure S4.** Composition of microbial communities at the Family level. **Figure S5.** Sample collection in the nine coastal regions of Southern China.

## Data Availability

Raw Illumina sequences were deposited in the National Center for Biotechnology Information (NCBI) and our SRA records will be accessible with the following link after the indicated release date: https://www.ncbi.nlm.nih.gov/sra/ SRP162721, SRA accession numbers: SRP162721.

## Abbreviations

F: female
HL: Hele county, Wanning city, Hainan province
HP: Hepu county, Behai city, Guangxi zhuang autonomous region
M: male
RA: Ruian county, Wenzhou city, Zhejiang province
*S. paramamosain*: *Scylla paramamosain*
SM: Sanmen county, Taizhou city, Zhejiang province
ST: Shantou city, Guangdong province
TS: Taishan county, Zhuhai city, Guangdong province
XP: Xiapu county, Ningde city, Fujian province
YJ: Yangjiang city, Guangdong province
YX: Yunxiao county, Zhangzhou city, Fujian province
