# Supplementary material for: High-throughput sequencing reveals the core gut microbiota of the mud crab (Scylla paramamosain) in different coastal regions of southern China
Source: BMC Genomics. 2019 Nov 8;20:829. doi: 10.1186/s12864-019-6219-7 (PMC6842235; doi:10.1186/s12864-019-6219-7)
Supplement: Supplementary file 1 — Additional file 1: Table S1. Number of bacterial taxonomic units. Table S2. Mean relative abundance of the 15 most abundant phyla in samples from the nine coastal regions of Southern China. Table S3. Mean relative abundance of the 15 most abundant genera in samples from the nine coastal regions of Southern China. Table S4. Salinity, temperature, pH, dissolved oxygen (DO), ammonia-nitrogen and nitrite levels of the nine regions of Southern China. Figure S1. Impact of sequencing depth and sampling on bacterial phylotypes. Figure S2. Composition of microbial communities at the Class level. Figure S3. Composition of microbial communities at the Order level. Figure S4. Composition of microbial communities at the Family level. Figure S5. Sample collection in the nine coastal regions of Southern China. [file 12864_2019_6219_MOESM1_ESM.docx]

**Supplementary information**

**Table S1. Number of bacterial taxonomic units**

| Sample ID | Number of taxonomic units | | | | |
| --- | --- | --- | --- | --- | --- |
|  | Phylum | Class | Order | Family | Genus |
| HL-F | 14±0.58 | 26±2.00 | 45±15.53 | 98±18.52 | 146±28.29 |
| HL-M | 25±4.58 | 44±6.81 | 98±8.50 | 195±16.50 | 344±36.12 |
| HP-F | 12±2.65 | 20±4.04 | 32±8.33 | 56±12.29 | 93±23.69 |
| HP-M | 18±6.08 | 33±9.85 | 68±27.02 | 126±61.52 | 227±117.63 |
| SM-F | 13±1.00 | 26±2.65 | 49±12.66 | 86±29.67 | 147±60.05 |
| SM-M | 17±2.52 | 31±4.04 | 55±8.33 | 90±18.25 | 147±39.15 |
| RA-F | 16±0.58 | 30±0.58 | 60±7.51 | 102±19.22 | 169±58.48 |
| RA-M | 13±0.58 | 22±1.53 | 36±4.58 | 56±8.02 | 96±18.15 |
| ST-F | 10±2.00 | 19±3.00 | 35±7.64 | 62±15.18 | 103±33.65 |
| ST-M | 18±2.00 | 32±4.16 | 65±16.26 | 129±35.23 | 228±49.67 |
| TS-F | 21±3.21 | 38±4.62 | 83±14.73 | 149±39.80 | 256±80.48 |
| TS-M | 14±4.62 | 24±8.96 | 44±20.22 | 75±43.47 | 114±58.53 |
| XP-F | 9±1.73 | 17±2.08 | 28±4.16 | 42±9.07 | 60±15.31 |
| XP-M | 9±1.00 | 18±2.31 | 27±6.24 | 40±11.14 | 60±23.71 |
| YJ-F | 20±7.21 | 36±12.86 | 77±35.55 | 152±76.41 | 270±138.39 |
| YJ-M | 18±2.00 | 31±2.08 | 63±1.15 | 111±9.50 | 183±18.50 |
| YX-F | 12±3.61 | 22±4.62 | 42±12.50 | 69±30.17 | 113±61.21 |
| YX-M | 15±0.58 | 32±1.15 | 65±2.00 | 118±1.00 | 215±3.61 |
| Total | 36 | 83 | 175 | 381 | 820 |
| Total-F | 30 | 67 | 148 | 322 | 711 |
| Total-M | 35 | 75 | 163 | 355 | 756 |

**Table S2. Mean relative abundance of the 15 most abundant phyla in samples from the nine coastal regions of Southern China**

| Samp  le ID | Mean relative abundance (%) at the phylum level | | | | | | | | | | | | | | |
| --- | --- | --- | --- | --- | --- | --- | --- | --- | --- | --- | --- | --- | --- | --- | --- |
|  | Tenericutes | Proteobacteria | Bacteroidetes | Firmicutes | Fusobacteria | Spirochaetae | Actinobacteria | Acidobacteria | Gemmatimonadetes | CKC4 | Deferribacteres | Cyanobacteria | Gracilibacteria | WCHB1_60 | Nitrospirae |
| HL-F | 32.47±13.80 | 23.16±13.52 | 28.48±20.38 | 2.82±2.13 | 7.62±5.04 | 5.18±7.05 | 0.10±0.04 | 0.05±0.05 | 0.01±0.01 | 0.04±0.03 | 0.00±0.00 | 0.00±0.00 | 0.00±0.01 | 0.01±0.00 | 0.00±0.01 |
| HL-M | 18.55±4.32 | 26.45±8.12 | 36.84±14.08 | 4.89±2.10 | 0.90±0.31 | 2.42±3.47 | 5.83±3.72 | 2.53±1.64 | 0.56±0.43 | 0.03±0.01 | 0.02±0.03 | 0.13±0.11 | 0.00±0.00 | 0.30±0.20 | 0.18±0.11 |
| HP-F | 52.01±2.29 | 9.13±1.97 | 19.73±5.11 | 11.97±9.85 | 2.15±0.37 | 4.70±1.92 | 0.01±0.00 | 0.00±0.00 | 0.00±0.00 | 0.28±0.28 | 0.00±0.00 | 0.00±0.00 | 0.00±0.00 | 0.00±0.00 | 0.00±0.00 |
| HP-M | 9.49±8.13 | 32.70±15.89 | 21.73±9.26 | 18.03±24.64 | 4.98±7.54 | 10.34±12.05 | 1.53±2.20 | 0.59±0.90 | 0.16±0.24 | 0.03±0.03 | 0.01±0.01 | 0.04±0.05 | 0.02±0.04 | 0.07±0.10 | 0.03±0.06 |
| SM -F | 31.58±24.10 | 16.39±4.41 | 24.02±8.18 | 14.72±23.07 | 9.66±9.01 | 2.89±4.70 | 0.47±0.76 | 0.046±0.07 | 0.00±0.00 | 0.03±0.03 | 0.05±0.08 | 0.05±0.05 | 0.05±0.03 | 0.00±0.00 | 0.00±0.00 |
| SM -M | 51.21±10.01 | 21.58±18.43 | 6.97±3.20 | 8.96±5.50 | 8.22±7.97 | 2.05±0.81 | 0.08±0.05 | 0.01±0.01 | 0.00±0.01 | 0.03±0.02 | 0.00±0.00 | 0.03±0.05 | 0.04±0.02 | 0.00±0.00 | 0.00±0.00 |
| RA-F | 28.14±18.23 | 13.83±0.94 | 20.28±11.25 | 15.13±14.84 | 19.92±7.20 | 1.81±2.15 | 0.28±0.28 | 0.09±0.08 | 0.01±0.01 | 0.04±0.03 | 0.05±0.08 | 0.17±0.29 | 0.02±0.02 | 0.00±0.00 | 0.01±0.01 |
| RA-M | 42.85±13.33 | 13.76±3.22 | 15.74±10.74 | 6.33±3.82 | 19.28±8.21 | 1.66±1.39 | 0.06±0.05 | 0.00±0.00 | 0.00±0.00 | 0.10±0.07 | 0.04±0.08 | 0.00±0.00 | 0.05±0.04 | 0.00±0.00 | 0.00±0.00 |
| ST-F | 60.99±25.80 | 17.90±19.64 | 7.77±7.54 | 9.12±8.17 | 3.25±4.28 | 0.85±0.97 | 0.06±0.10 | 0.00±0.00 | 0.00±0.00 | 0.01±0.00 | 0.00±0.10 | 0.00±0.00 | 0.01±0.01 | 0.00±0.00 | 0.00±0.00 |
| ST-M | 12.15±16.25 | 36.26±17.57 | 13.14±17.88 | 23.79±19.36 | 12.74±20.69 | 0.30±0.49 | 0.72±0.36 | 0.16±0.18 | 0.03±0.03 | 0.03±0.03 | 0.48±0.83 | 0.01±0.01 | 0.02±0.01 | 0.01±0.10 | 0.02±0.01 |
| TS-F | 46.23±11.18 | 24.39±8.56 | 10.39±8.30 | 8.16±5.21 | 7.91±11.30 | 0.00±0.00 | 1.51±1.12 | 0.69±0.46 | 0.10±0.08 | 0.33±0.51 | 0.00±0.00 | 0.04±0.04 | 0.01±0.01 | 0.07±0.06 | 0.03±0.02 |
| TS-M | 7.39±1.69 | 50.21±12.30 | 10.35±13.72 | 18.15±12.97 | 13.25±8.40 | 0.00±0.01 | 0.13±0.19 | 0.07±0.11 | 0.01±0.02 | 0.13±0.08 | 0.00±0.00 | 0.01±0.01 | 0.20±0.12 | 0.00±0.10 | 0.00±0.01 |
| XP-F | 49.65±5.73 | 31.53±12.03 | 4.76±5.76 | 5.89±4.01 | 7.48±11.04 | 0.58±0.99 | 0.01±0.01 | 0.00±0.00 | 0.00±0.00 | 0.02±0.02 | 0.00±0.00 | 0.00±0.00 | 0.01±0.01 | 0.00±0.00 | 0.00±0.00 |
| XP-M | 43.87±32.24 | 29.52±16.73 | 7.36±11.73 | 6.89±4.22 | 9.24±12.46 | 3.05±4.98 | 0.02±0.02 | 0.00±0.00 | 0.00±0.00 | 0.04±0.05 | 0.00±0.00 | 0.00±0.00 | 0.00±0.00 | 0.00±0.00 | 0.00±0.00 |
| YJ-F | 1.90±0.72 | 25.12±12.34 | 37.36±16.07 | 5.83±3.15 | 4.61±4.13 | 15.74±18.21 | 5.60±8.49 | 2.36±3.55 | 0.50±0.78 | 0.07±0.10 | 0.00±0.01 | 0.12±0.17 | 0.18±0.17 | 0.25±0.37 | 0.00±0.21 |
| YJ-M | 5.93±4.89 | 41.83±22.38 | 21.87±14.13 | 21.58±20.05 | 4.74±3.76 | 1.97±2.38 | 1.07±0.55 | 0.18±0.25 | 0.06±0.06 | 0.11±0.15 | 0.11±0.10 | 0.07±0.07 | 0.05±0.05 | 0.01±0.10 | 0.00±0.02 |
| YX-F | 7.85±8.54 | 20.81±7.04 | 21.07±9.96 | 21.43±23.31 | 19.06±15.92 | 9.08±7.66 | 0.42±0.73 | 0.02±0.03 | 0.00±0.00 | 0.05±0.03 | 0.05±0.09 | 0.02±0.03 | 0.05±0.02 | 0.00±0.00 | 0.00±0.11 |
| YX-M | 0.97±0.16 | 22.98±7.63 | 31.25±2.89 | 42.23±5.53 | 0.59±0.13 | 0.06±0.03 | 1.22±0.28 | 0.12±0.06 | 0.06±0.06 | 0.03±0.01 | 0.10±0.10 | 0.15±0.03 | 0.05±0.06 | 0.00±0.00 | 0.00±0.05 |

**Table S3. Mean relative abundance of the 15 most abundant genera in samples from the nine coastal regions of Southern China**

| Sample ID | Mean relative abundance (%) at the genu level | | | | | | | | | | | | | | |
| --- | --- | --- | --- | --- | --- | --- | --- | --- | --- | --- | --- | --- | --- | --- | --- |
|  | Candidatus_  Hepatoplasma | Arcobacter | Photobacterium | Vibrio | Carboxylicivirga | Bacteroides | Spirochaeta_2 | Psychrilyobacter | Sunxiuqinia | Parabacteroides | Defluviitalea  ceae_UCG_011 | Lachnoclostridium | Shewanella | Enterococcus | Clostridium_s  ensu_stricto_11 |
| HL-F | 6.62±11.10 | 8.76±5.26 | 2.14±1.00 | 10.33±7.29 | 11.69±15.41 | 11.04±16.09 | 5.18±7.05 | 0.01±0.02 | 4.61±7.55 | 0.00±0.00 | 0.95±0.77 | 0.00±0.00 | 0.80±0.14 | 0.00±0.00 | 0.13±0.13 |
| HL-M | 0.25±0.21 | 3.41±2.35 | 2.48±2.51 | 5.69±5.83 | 6.63±5.06 | 5.82±9.22 | 2.41±3.48 | 0.12±0.16 | 14.18±11.19 | 0.02±0.02 | 0.49±0.61 | 0.02±0.01 | 1.21±1.29 | 0.14±0.04 | 0.14±0.23 |
| HP-F | 32.12±15.15 | 3.05±2.30 | 0.74±0.36 | 2.41±0.32 | 2.79±1.47 | 0.98±0.74 | 4.70±1.92 | 0.81±0.70 | 14.12±6.80 | 0.00±0.01 | 1.75±1.86 | 0.01±0.01 | 0.66±0.21 | 0.00±0.00 | 0.07±0.04 |
| HP-M | 0.84±0.46 | 7.24±6.10 | 3.96±4.21 | 9.82±9.40 | 3.90±3.42 | 0.12±0.06 | 10.33±12.04 | 0.00±0.00 | 4.60±4.18 | 10.26±17.72 | 0.25±0.43 | 12.83±22.18 | 0.88±1.23 | 0.16±0.24 | 0.21±0.27 |
| SM -F | 22.64±18.62 | 2.12±1.96 | 6.78±7.06 | 0.91±0.91 | 2.08±3.29 | 2.32±3.76 | 2.89±4.70 | 4.53±3.28 | 0.08±0.08 | 3.13±5.41 | 0.21±0.21 | 0.77±1.33 | 0.02±0.01 | 2.16±3.73 | 0.01±0.01 |
| SM -M | 22.11±19.48 | 5.44±2.70 | 13.58±18.94 | 0.88±0.26 | 1.41±1.39 | 0.35±0.34 | 2.05±0.81 | 5.86±6.05 | 2.17±2.01 | 0.01±0.02 | 4.83±7.03 | 0.03±0.04 | 0.38±0.12 | 0.01±0.02 | 1.32±0.63 |
| RA-F | 19.78±15.85 | 4.91±3.64 | 3.51±3.09 | 0.69±0.28 | 4.30±1.76 | 1.20±2.04 | 1.81±2.15 | 18.87±7.77 | 0.46±0.65 | 1.83±3.17 | 3.03±2.19 | 0.64±1.09 | 0.05±0.03 | 1.10±1.91 | 0.19±0.06 |
| RA-M | 24.66±18.57 | 5.50±1.76 | 5.60±3.56 | 0.62±0.18 | 5.44±4.86 | 0.08±0.12 | 1.66±1.39 | 16.49±5.60 | 1.45±1.16 | 0.02±0.03 | 3.18±0.97 | 0.00±0.00 | 0.11±0.10 | 0.00±0.00 | 0.63±0.55 |
| ST-F | 31.95±20.71 | 3.11±3.18 | 5.16±4.46 | 5.60±9.48 | 3.34±2.69 | 3.56±5.51 | 0.85±0.97 | 2.61±4.50 | 0.00±0.00 | 0.17±0.29 | 2.18±1.49 | 0.00±0.01 | 1.38±2.10 | 0.00±0.00 | 0.81±1.40 |
| ST-M | 6.99±12.04 | 9.25±8.01 | 4.21±4.83 | 10.78±10.94 | 3.28±4.32 | 0.41±0.64 | 0.29±0.49 | 0.01±0.01 | 0.00±0.00 | 0.46±0.79 | 0.47±0.76 | 0.20±0.34 | 1.87±2.25 | 0.39±0.67 | 1.68±1.24 |
| TS-F | 41.60±10.48 | 15.66±11.52 | 0.00±0.00 | 4.16±5.84 | 0.70±0.49 | 5.38±4.94 | 0.00±0.00 | 0.00±0.00 | 0.00±0.00 | 0.05±0.09 | 0.00±0.01 | 0.04±0.06 | 0.27±0.30 | 0.02±0.01 | 0.27±0.24 |
| TS-M | 4.53±1.97 | 30.25±14.33 | 0.01±0.01 | 6.39±5.22 | 0.17±0.27 | 1.24±1.98 | 0.00±0.01 | 0.00±0.00 | 0.00±0.00 | 0.00±0.00 | 0.05±0.07 | 0.00±0.00 | 5.22±1.85 | 0.01±0.01 | 2.04±0.26 |
| XP-F | 46.37±7.63 | 3.10±2.71 | 26.17±15.03 | 1.65±1.45 | 1.91±1.29 | 0.00±0.00 | 0.58±0.99 | 2.19±3.78 | 0.23±0.40 | 0.00±0.00 | 2.16±1.84 | 0.00±0.00 | 0.11±0.09 | 0.00±0.00 | 2.67±3.07 |
| XP-M | 39.19±34.33 | 4.15±2.69 | 24.46±18.08 | 0.48±0.32 | 0.83±0.49 | 0.01±0.02 | 3.05±4.98 | 6.00±10.39 | 0.01±0.01 | 0.00±0.00 | 3.52±3.89 | 0.00±0.00 | 0.08±0.09 | 0.00±0.00 | 2.47±3.58 |
| YJ-F | 0.00±0.00 | 7.56±7.64 | 5.27±3.93 | 0.41±0.57 | 8.92±11.13 | 20.47±23.20 | 15.73±18.21 | 0.00±0.00 | 0.27±0.29 | 0.04±0.05 | 0.63±1.07 | 0.04±0.01 | 0.08±0.09 | 0.13±0.15 | 0.30±0.52 |
| YJ-M | 0.33±0.40 | 5.54±6.54 | 12.03±18.13 | 13.71±11.60 | 5.63±8.12 | 3.13±2.47 | 1.97±2.38 | 0.09±0.10 | 0.24±0.24 | 3.10±4.81 | 0.28±0.44 | 1.41±1.67 | 1.95±3.13 | 1.94±3.24 | 0.03±0.05 |
| YX-F | 3.35±2.16 | 4.60±3.98 | 5.28±5.02 | 1.33±0.85 | 7.32±5.85 | 5.02±5.22 | 9.08±7.66 | 0.02±0.03 | 0.01±0.02 | 3.05±5.29 | 3.41±5.48 | 1.10±1.97 | 0.09±0.08 | 2.08±3.55 | 0.24±0.26 |
| YX-M | 0.74±0.16 | 0.38±0.33 | 0.89±0.36 | 4.70±7.78 | 0.54±0.49 | 5.43±0.80 | 0.05±0.04 | 0.15±0.09 | 0.07±0.09 | 8.34±1.15 | 0.15±0.22 | 1.85±0.17 | 0.17±0.19 | 5.16±0.38 | 0.00±0.00 |

**Table S4. Salinity, temperature, pH, dissolved oxygen (DO), ammonia-nitrogen and nitrite levels of the nine regions of Southern China**

| Month | Region | Salinity | Temperature (℃) | pH | DO (mg/L) | Ammonia-nitrogen (mg/L) | Nitrite (mg/L) |
| --- | --- | --- | --- | --- | --- | --- | --- |
|  | HL | 16.25 ± 2.32 | 27.17 ± 3.24 | 8.15 ± 0.27 | 8.42 ± 0.77 | 0.56 ± 0.09 | 0.12 ± 0.04 |
|  | HP | 15.14 ± 2.11 | 27.32 ± 3.84 | 8.16 ± 0.24 | 8.16 ± 0.86 | 0.66 ± 0.08 | 0.13 ± 0.04 |
|  | YJ | 15.13 ± 2.13 | 27.32 ± 2.68 | 8.12 ± 0.22 | 8.46 ± 0.69 | 0.53 ± 0.10 | 0.11 ± 0.03 |
|  | TS | 14.56 ± 2.42 | 26.26 ± 2.49 | 8.10 ± 0.21 | 8.32 ± 0.63 | 0.57 ± 0.09 | 0.12 ± 0.04 |
| May | ST | 15.23 ± 2.12 | 26.16 ± 2.37 | 8.16 ± 0.12 | 8.29 ± 0.77 | 0.60 ± 0.08 | 0.13 ± 0.03 |
|  | YX | 16.20 ± 2.34 | 26.20 ± 2.13 | 8.19 ± 0..11 | 8.14 ± 0.84 | 0.63 ± 0.10 | 0.12 ± 0.03 |
|  | XP | 17.13 ± 1.70 | 26.11 ± 2.59 | 8.13 ± 0.14 | 8.09 ± 0.93 | 0.57 ± 0.08 | 0.14 ± 0.03 |
|  | RA | 18.25 ± 1.81 | 26.05 ± 2.80 | 8.25 ± 0.12 | 8.01 ± 0.89 | 0.57 ± 0.09 | 0.12 ± 0.03 |
|  | SM | 17.11 ± 1.61 | 25.72 ± 2.63 | 8.11 ± 0.15 | 8.25 ± 0.84 | 0.52 ± 0.11 | 0.11 ± 0.05 |
|  | HL | 15.23 ± 2.38 | 28.85 ± 4.26 | 8.25 ± 0.14 | 7.56 ± 0.92 | 0.79 ± 0.12 | 0.13 ± 0.03 |
|  | HP | 14.02 ± 2.24 | 28.71 ± 3.43 | 8.14 ± 0.16 | 7.59 ± 0.91 | 0.78 ± 0.12 | 0.14 ± 0.04 |
|  | YJ | 14.05 ± 2.16 | 28.05 ± 3.28 | 8.18 ± 0.12 | 7.63 ± 0.89 | 0.79 ± 0.10 | 0.11 ± 0.04 |
|  | TS | 14.68 ± 2.48 | 27.05 ± 4.37 | 8.23 ± 0.17 | 7.54 ± 0.94 | 0.76 ± 0.09 | 0.13 ± 0.02 |
| June | ST | 15.12 ± 2.42 | 27.15 ± 3.94 | 8.24 ± 0.10 | 7.60 ± 0.86 | 0.78 ± 0.10 | 0.12 ± 0.03 |
|  | YX | 15.18 ± 2.38 | 27.09 ± 3.42 | 8.19 ± 0.13 | 7.55 ± 0.96 | 0.79 ± 0.10 | 0.13 ± 0.03 |
|  | XP | 16.13 ± 2.05 | 27.29 ±2.89 | 8.14 ± 0.11 | 7.49 ± 0.93 | 0.76 ± 0.12 | 0.14 ± 0.03 |
|  | RA | 16.25 ± 1.98 | 27.10 ±3.01 | 8.23 ± 0.11 | 7.66 ± 0.95 | 0.77 ± 0.11 | 0.13 ±0.05 |
|  | SM | 17.01 ± 2.02 | 27.21 ±3.13 | 8.23 ± 0.13 | 7.26 ± 0.87 | 0.76 ± 0.19 | 0.14 ± 0.06 |
|  | HL | 13.22 ± 2.13 | 28.25 ± 3.76 | 8.17 ± 0.14 | 5.29 ± 0.89 | 0.81 ± 0.06 | 0.13 ± 0.02 |
|  | HP | 13.56 ± 2.02 | 27.96 ± 5.21 | 8.13 ± 0.12 | 5.62 ± 0.88 | 0.75 ± 0.07 | 0.12 ± 0.03 |
|  | YJ | 12.97 ± 2.22 | 27.64± 4.94 | 8.19 ± 0.16 | 5.34 ± 0.89 | 0.74 ± 0.09 | 0.13 ± 0.03 |
|  | TS | 13.45 ± 2.05 | 27.53 ± 3.76 | 8.20 ± 0.18 | 5.57 ± 0.94 | 0.75 ± 0.09 | 0.13 ± 0.02 |
| July | ST | 13.39 ± 2.08 | 27.45 ± 3.58 | 8.01 ± 0.20 | 5.63 ± 0.79 | 0.77 ± 0.08 | 0.12 ± 0.04 |
|  | YX | 13.50 ± 2.02 | 27.31 ± 3.67 | 8.07 ± 0.21 | 5.19 ± 0.85 | 0.79 ± 0.08 | 0.14 ± 0.03 |
|  | XP | 15.46 ± 1.66 | 27.24 ± 3.81 | 8.22 ± 0.16 | 5.20 ± 0.97 | 0.75 ± 0.08 | 0.13 ± 0.03 |
|  | RA | 15.61 ± 1.88 | 27.18 ± 3.99 | 8.19 ± 0.13 | 5.26 ± 0.96 | 0.76 ± 0.08 | 0.12 ± 0.04 |
|  | SM | 15.81 ± 1.76 | 27.38 ± 3.87 | 8.05 ± 0.25 | 5.17 ± 0.87 | 0.79 ± 0.09 | 0.12 ± 0.05 |

**
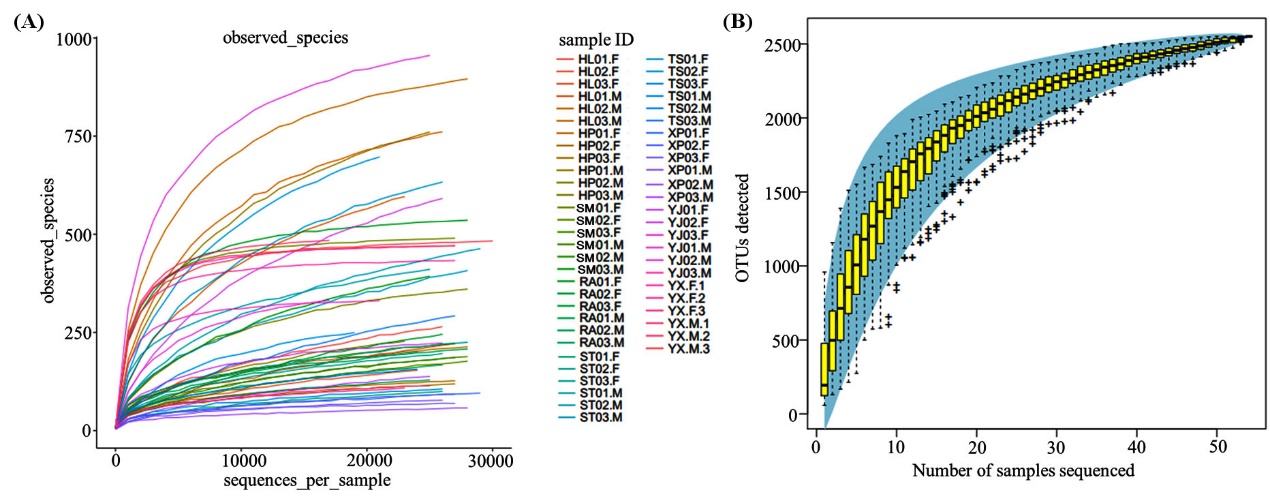
**

**Figure S1. Impact of sequening deth and sampling on bacterial phylotypes detection.**


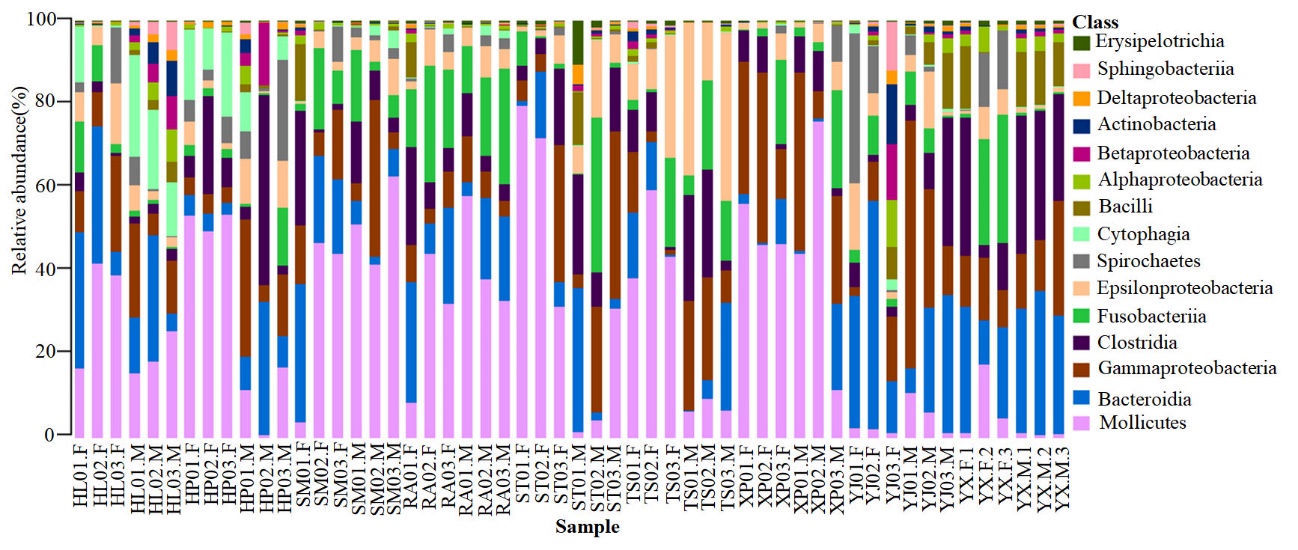


**Figure S2. Composition of Microbial community at Class level.**

**
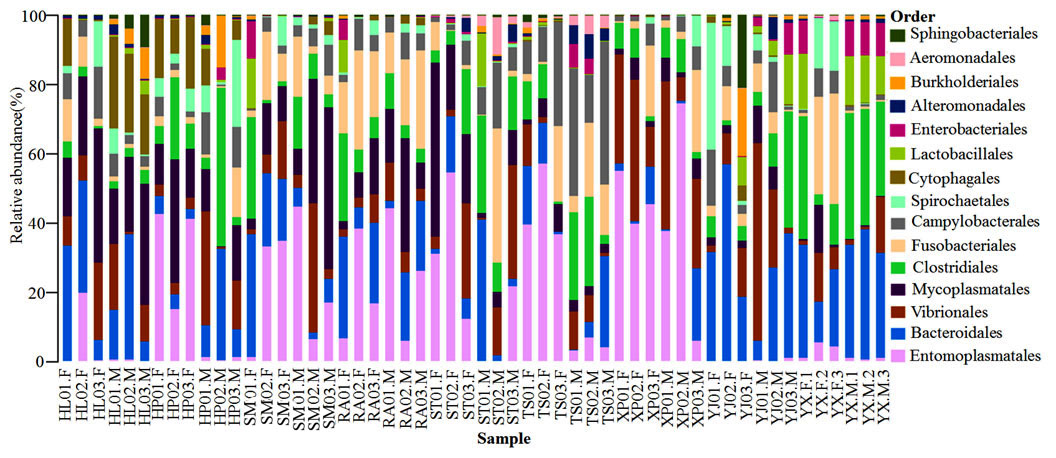
**

**Figure S3. Composition of Microbial community at Order level.**

**
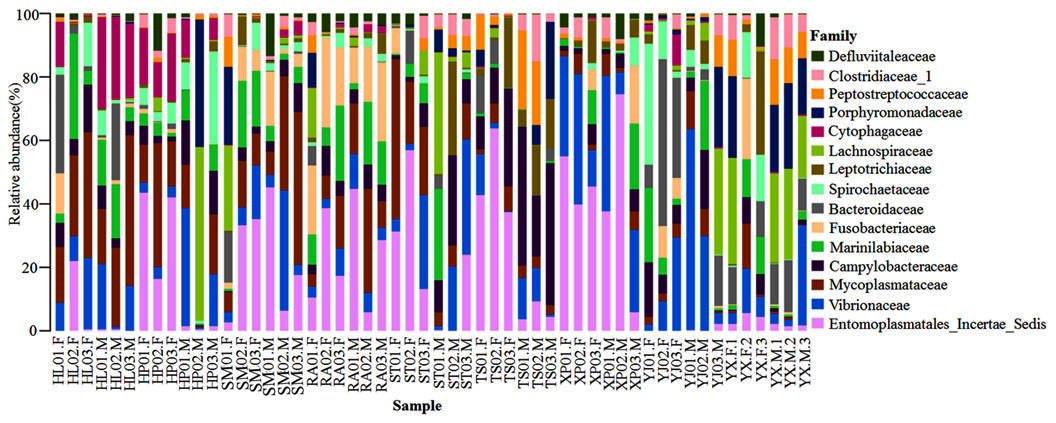
**

**Figure S4. Composition of Microbial community at Family level.**

**
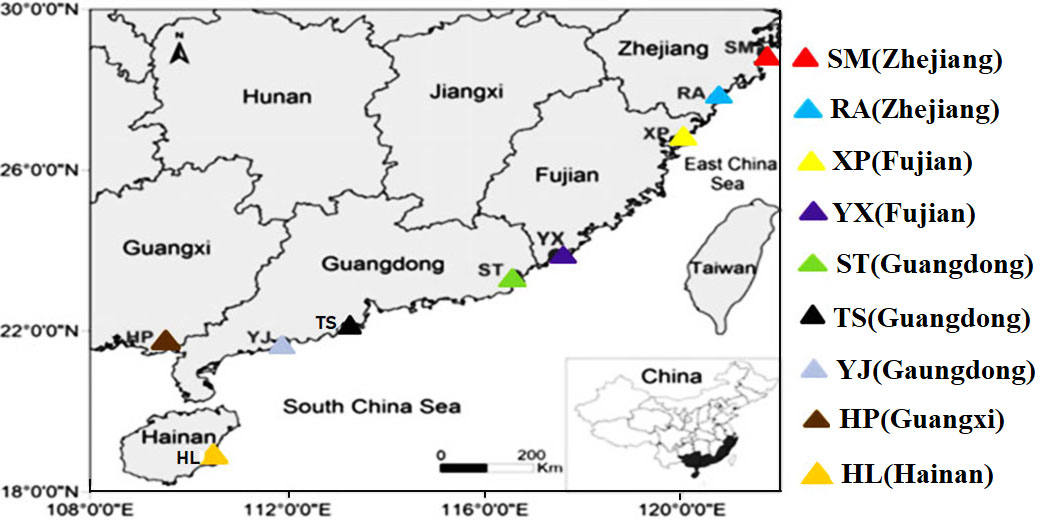
**

**Figure S5. Samples collection in nine regions of the coasts of southern China**
